# Supplementary material for: Pathophysiological Aspects of Muscle Atrophy and Osteopenia Induced by Chronic Constriction Injury (CCI) of the Sciatic Nerve in Rats
Source: Int J Mol Sci. 2023 Feb 13;24(4):3765. doi: 10.3390/ijms24043765 (PMC9962869; doi:10.3390/ijms24043765)
Supplement: Supplementary file 1 [file ijms-24-03765-s001.zip › ijms-2190555-supplementary.pdf]

Original Western Blots (corresponding to Figure 7A in the main article). Chemiluminescence was detected using the Amersham ECL kit (Amersham, Buckingham, UK) and the Uvitec Alliance 6.7-01 Western Blot imaging system (Uvitec, Cambridge, UK).

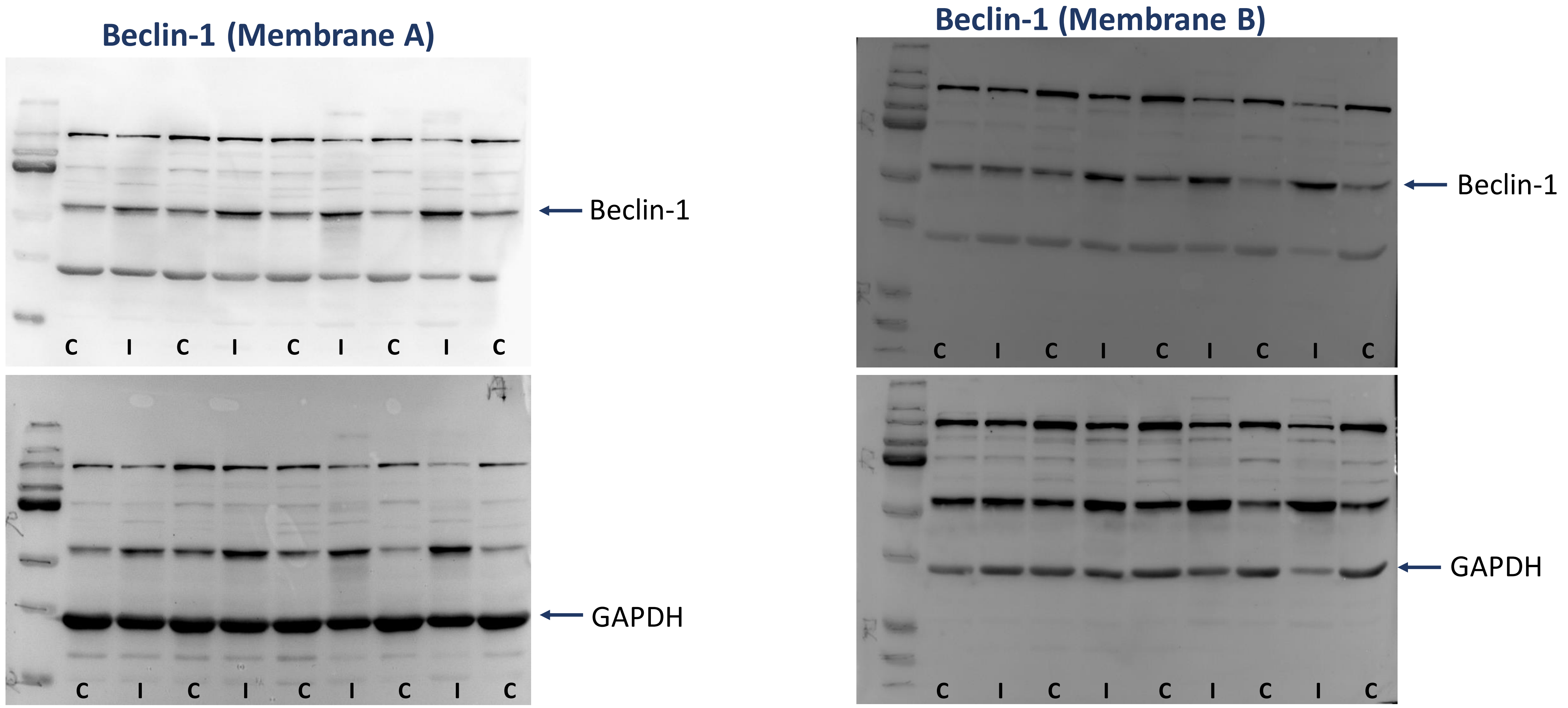

The top membranes were probed for Beclin-1 (Cell Signaling 3738S; Diluition 1:1000), the lower membranes for GAPDH (Antibody: Abcam Ab181602 ; Diluition 1: 40.000). Sample order is as shown at the bottom of the membrane (C=contra; I=Ipsi).

Original Western Blots (corresponding to Figure 7B in the main article). Chemiluminescence was detected using the Amersham ECL kit (Amersham, Buckingham, UK) and the Uvitec Alliance 6.7-01 Western Blot imaging system (Uvitec, Cambridge, UK).

LC3B I/LC3B II (Membrane A)

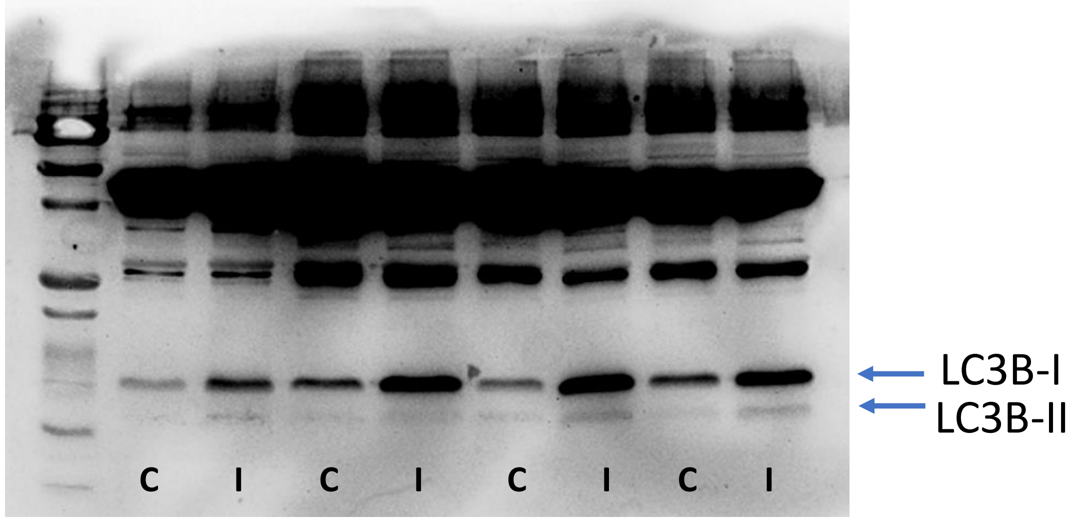

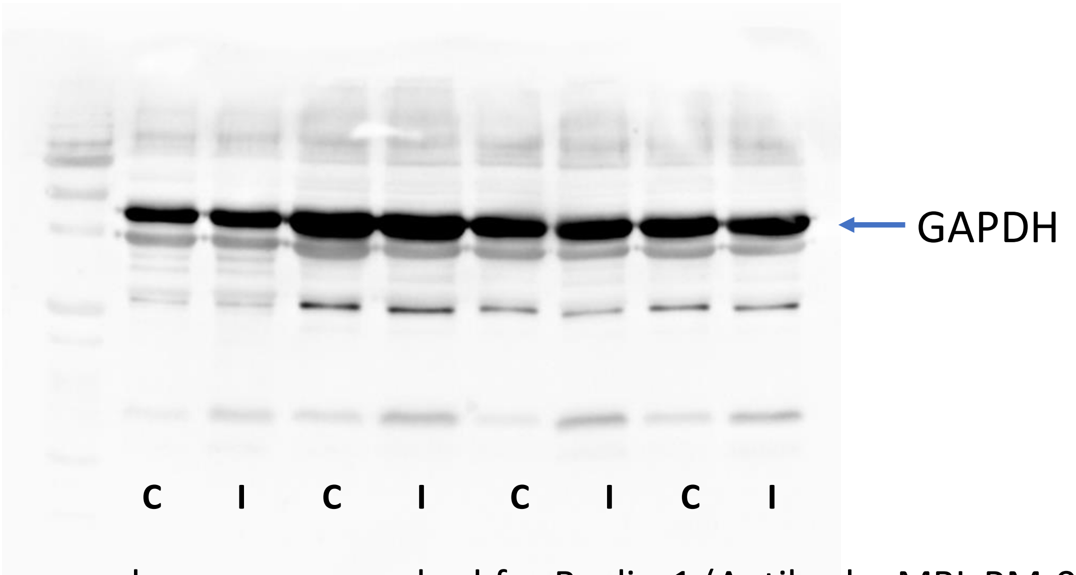

LC3B I/LC3B II (Membrane B)

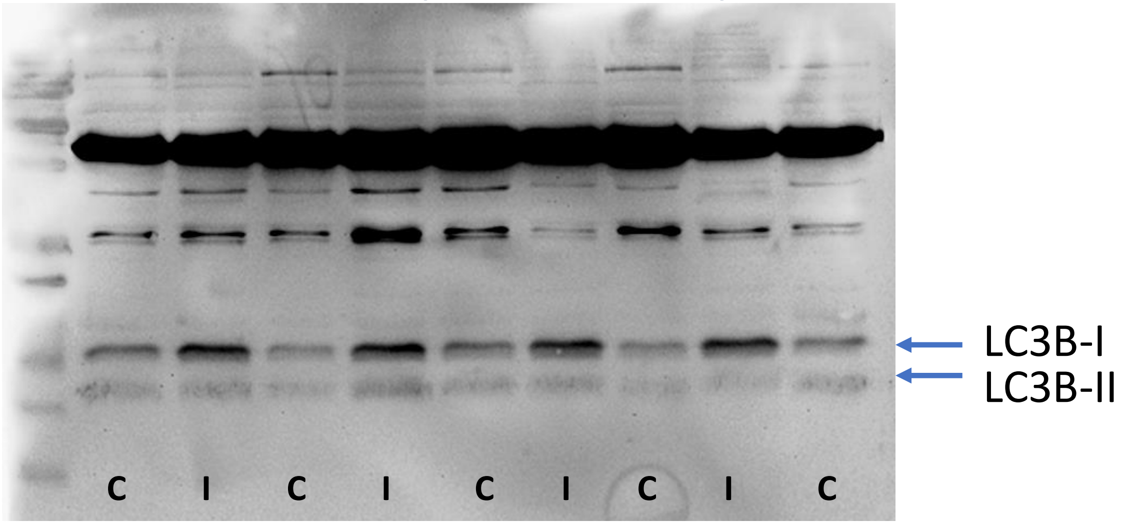

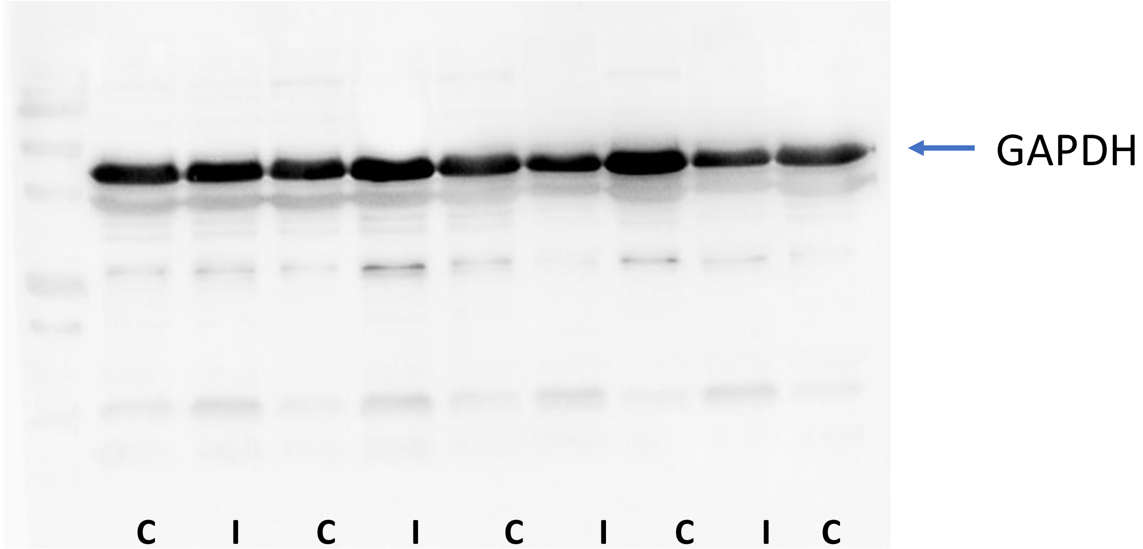

The top membranes were probed for Beclin-1 (Antibody: MBL PM-036; Diluition 1:1000), the lower membranes for GAPDH (Antibody: Abcam Ab181602 ; Diluition 1: 40.000). Sample order is as shown at the bottom of the membrane (C=contra; I=Ipsi).

Original Western Blots (corresponding to Figure 7C in the main article). Chemiluminescence was detected using the Amersham ECL kit (Amersham, Buckingham, UK) and the Uvitec Alliance 6.7-01 Western Blot imaging system (Uvitec, Cambridge, UK).

p62 (Membrane A)

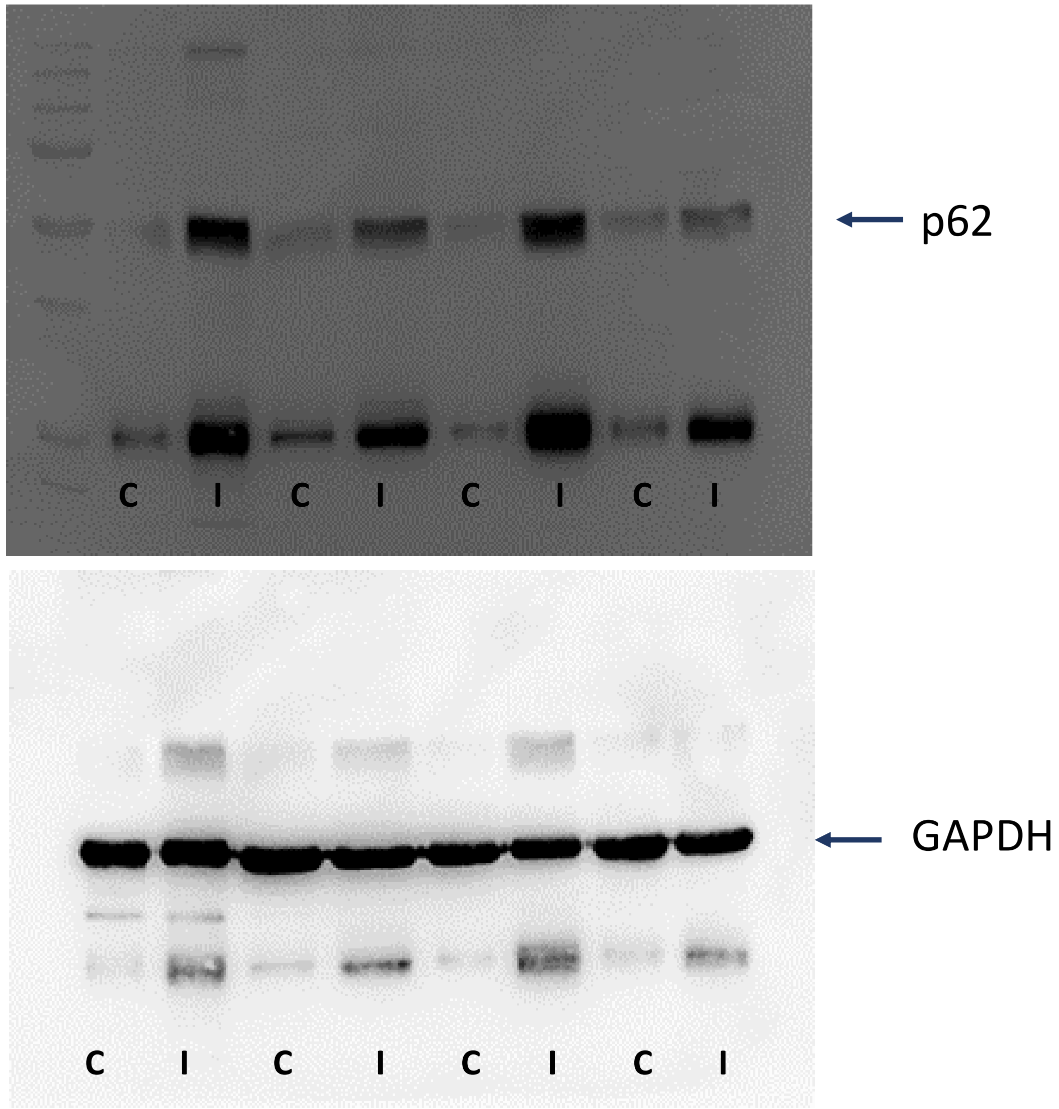

p62 (Membrane B)

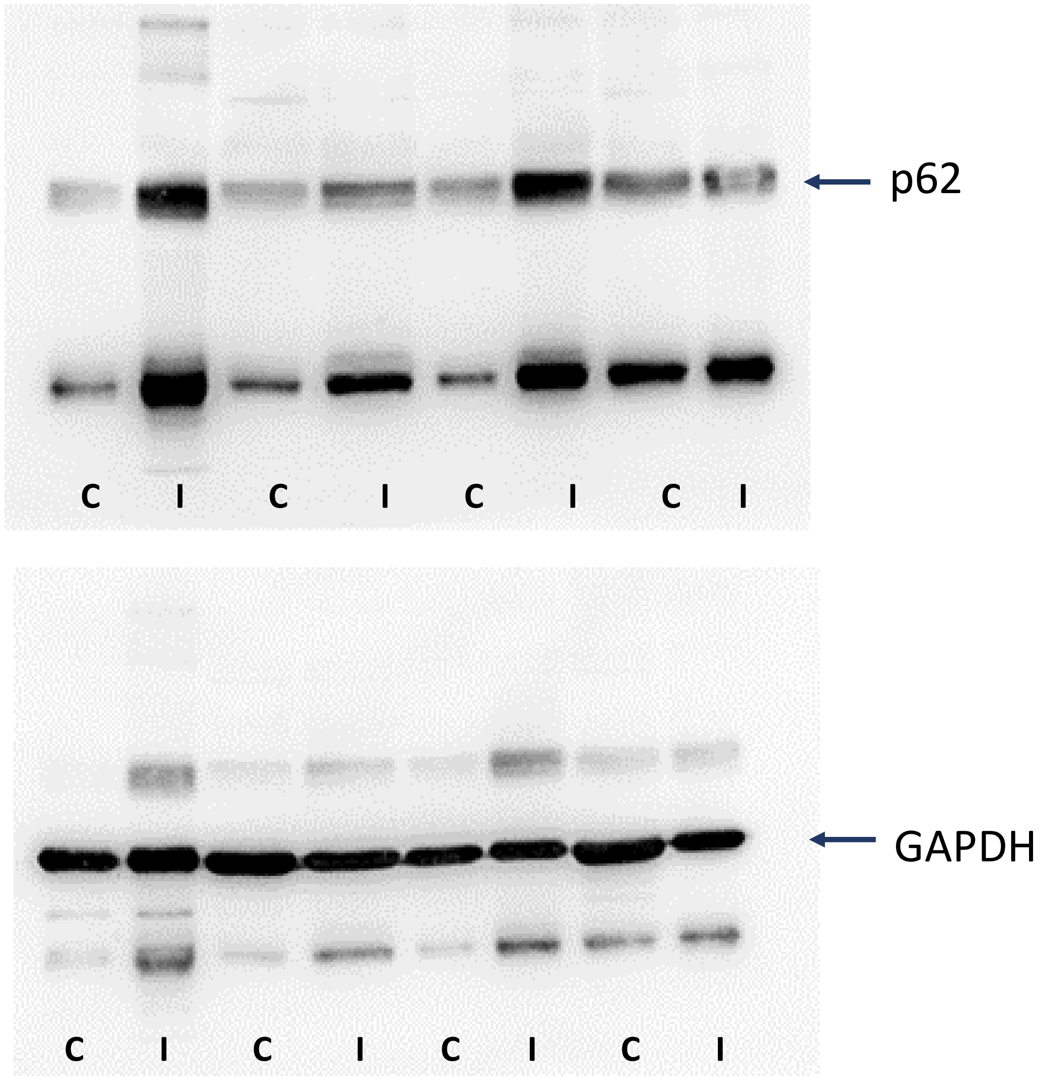

The top membranes were probed for p62 (Antibody: MBL M162-3; Diluition 1:1000), the lower membranes for GAPDH (Antibody: Abcam Ab181602 ; Diluition 1: 40.000). Sample order is as shown at the bottom of the membrane (C=contra; I=Ipsi).

Original Western Blots (corresponding to Figure 7D in the main article). Chemiluminescence was detected using the Amersham ECL kit (Amersham, Buckingham, UK) and the Uvitec Alliance 6.7-01 Western Blot imaging system (Uvitec, Cambridge, UK).

TRAF-6 (Membrane A)

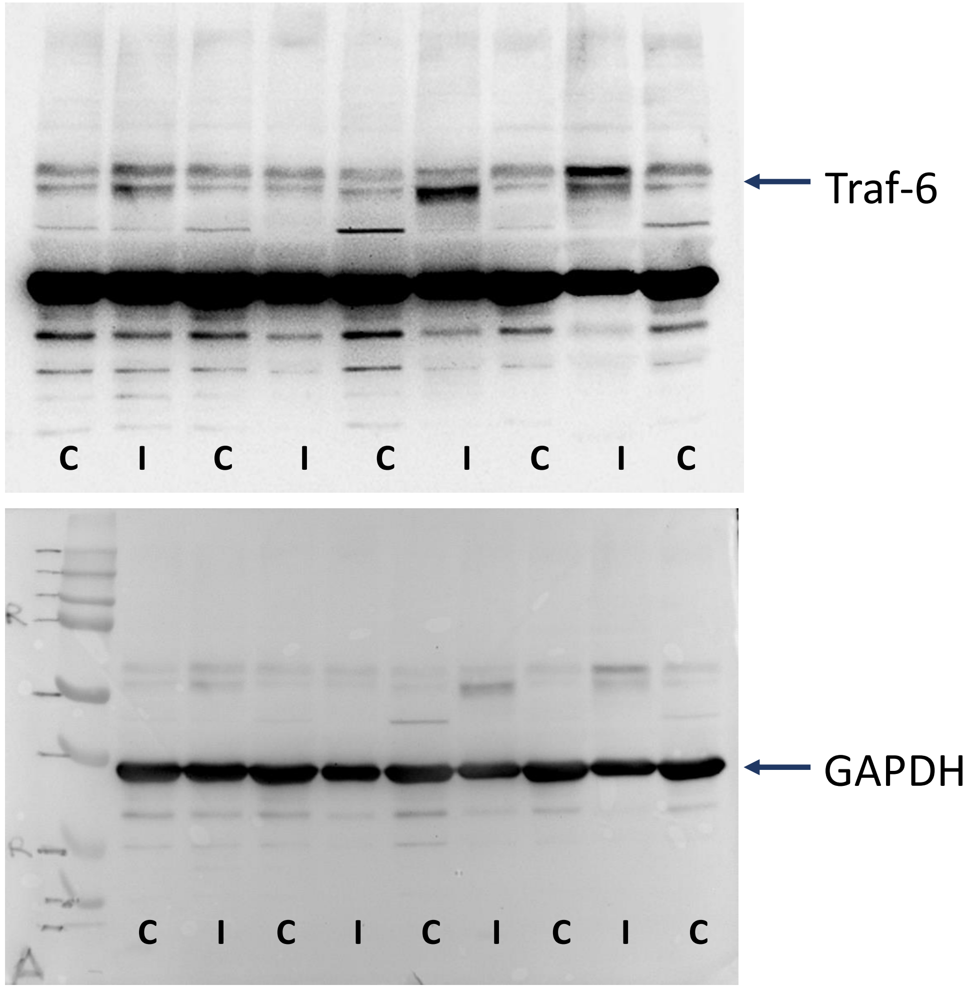

TRAF-6 (Membrane B)

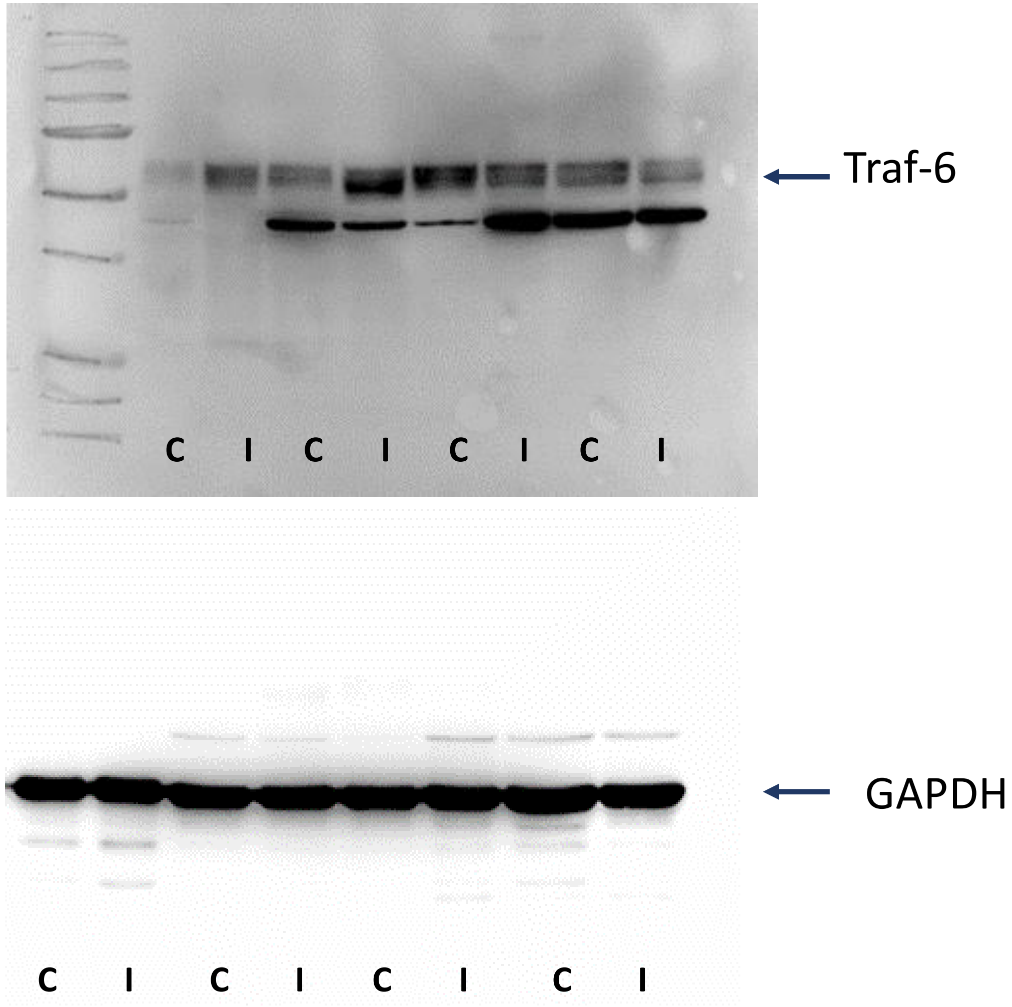

The top membranes were probed for TRAF-6 (Antibody: Abcam Ab33915; Diluition 1:1000), the lower membranes for GAPDH (Antibody: Abcam Ab181602 ; Diluition 1: 40.000). Sample order is as shown at the bottom of the membrane (C=contra; I=Ipsi).

Original Western Blots (corresponding to Figure 7E in the main article). Chemiluminescence was detected using the Amersham ECL kit (Amersham, Buckingham, UK) and the Uvitec Alliance 6.7-01 Western Blot imaging system (Uvitec, Cambridge, UK).

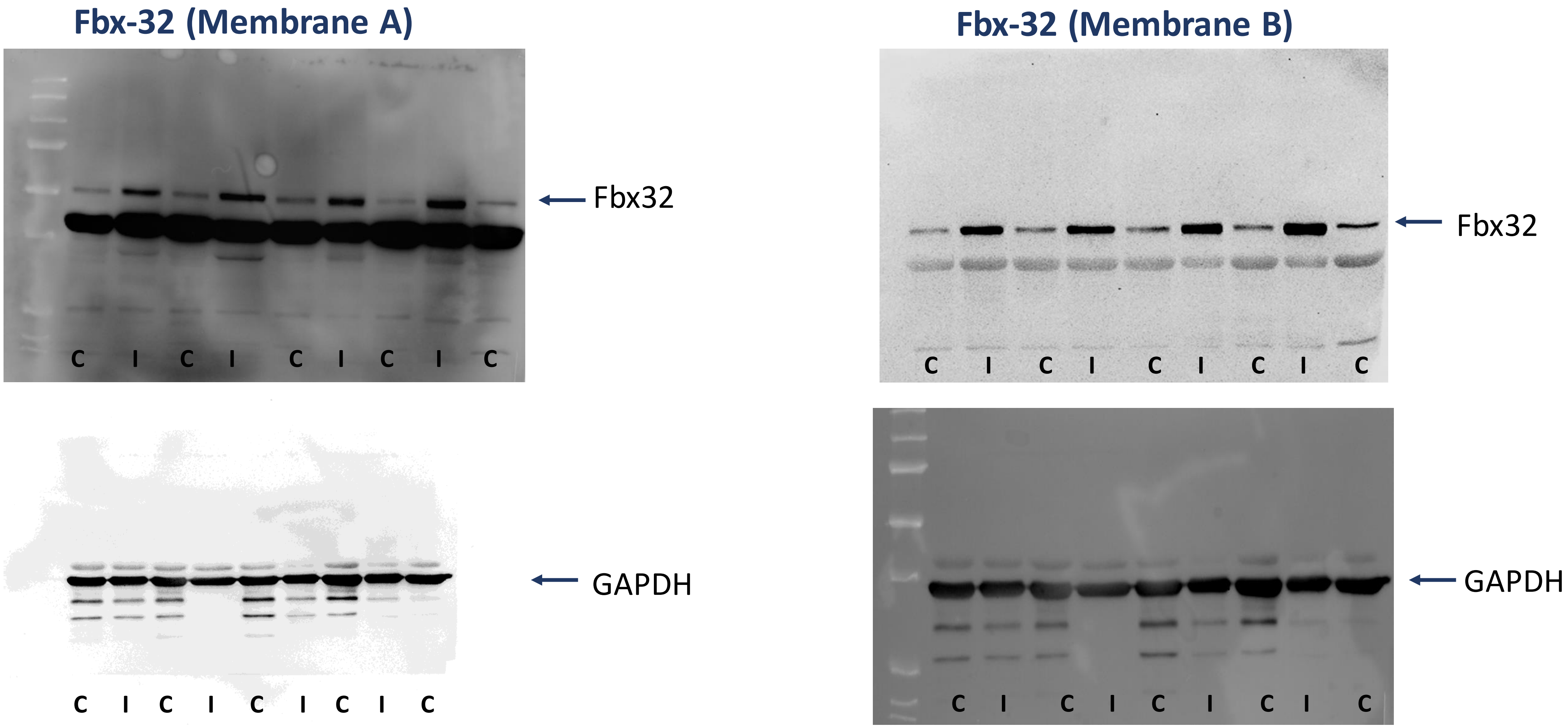

The top membranes were probed for Fbx32 (Antibody: Abcam Ab 168372; Diluition 1:1000), the lower membranes for GAPDH (Antibody: Abcam Ab 168372; Diluition 1:1000). Sample order is as shown at the bottom of the membrane (C=contra; I=Ipsi).

Original Western Blots (corresponding to Figure 7F in the main article). Chemiluminescence was detected using the Amersham ECL kit (Amersham, Buckingham, UK) and the Uvitec Alliance 6.7-01 Western Blot imaging system (Uvitec, Cambridge, UK).

Pax-7 (Membrane A)

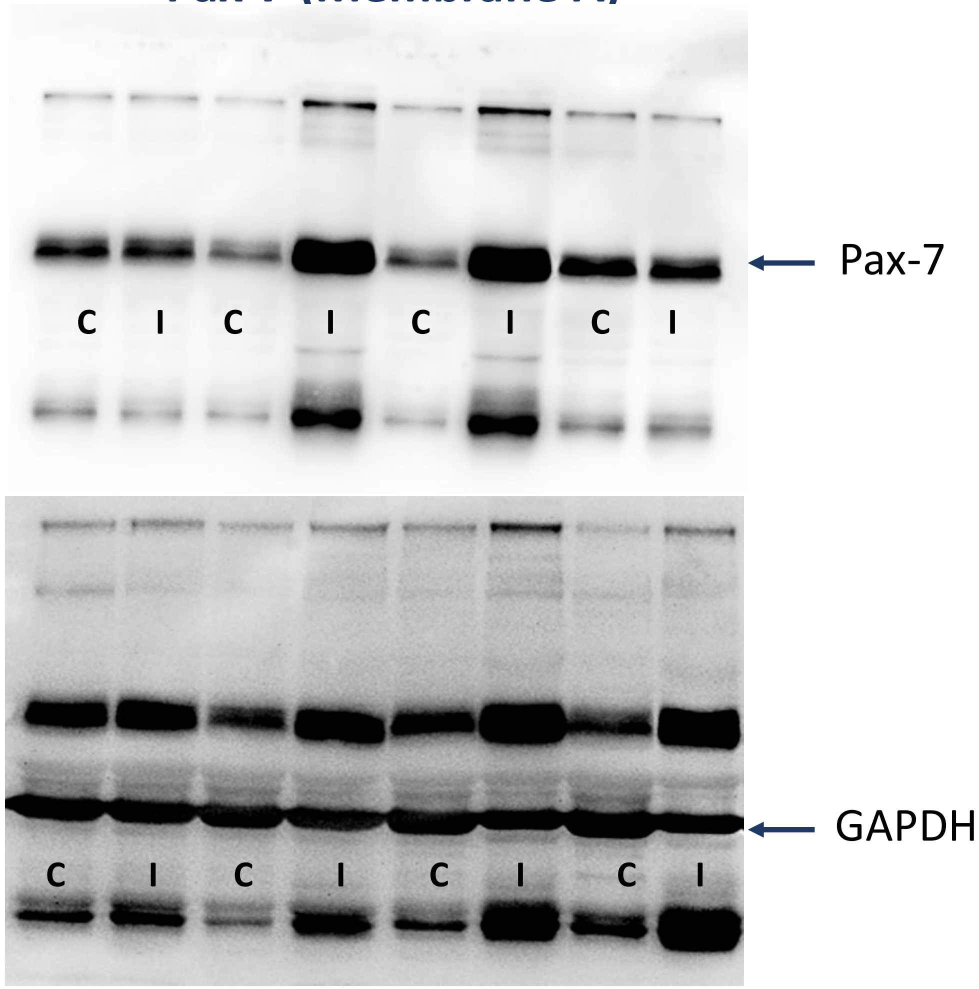

Pax-7 (Membrane B)

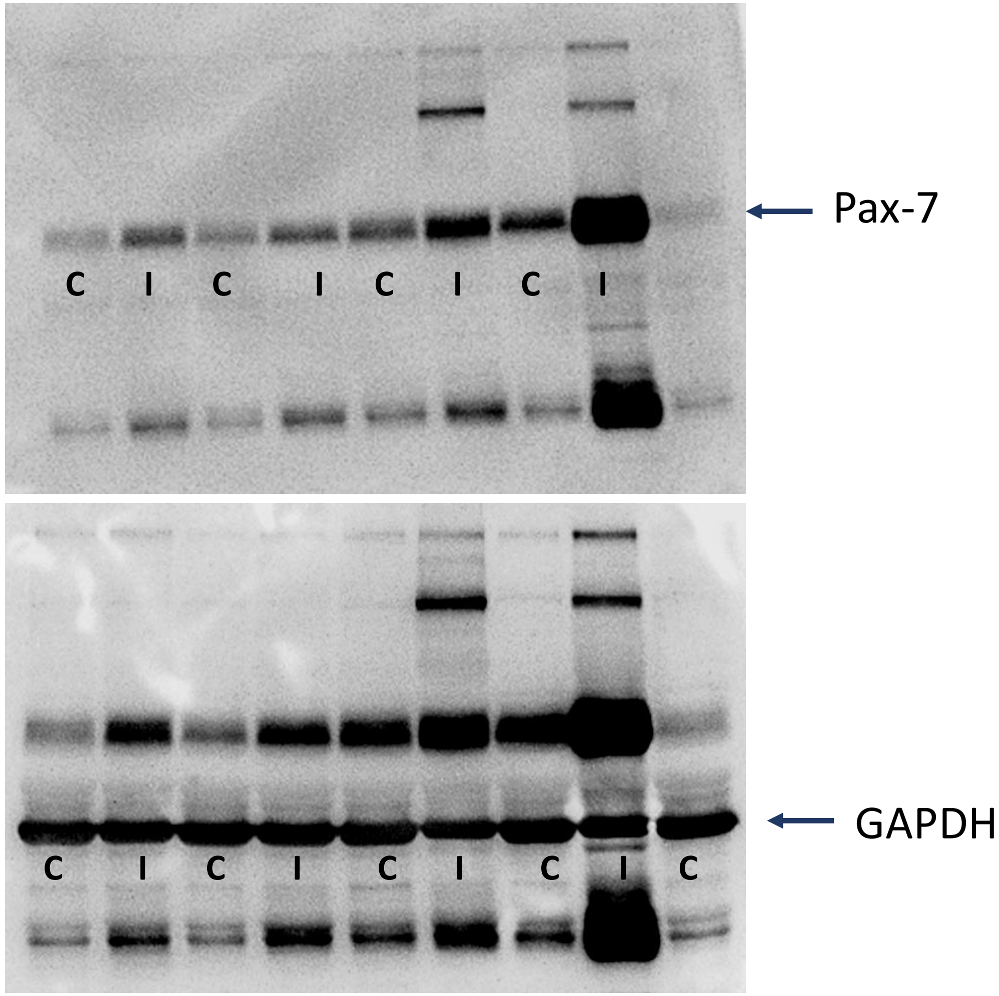

The top membranes were probed for Pax-7 (Antibody Merk Millipore, MABD20; Diluition 1:1000), the lower membranes for GAPDH (Antibody: Abcam Ab181602 ; Diluition 1: 40.000). Sample order is as shown at the bottom of the membrane (C=contra; I=Ipsi).
